# School Closure to Reduce Influenza Transmission

**DOI:** 10.3201/eid1501.081289

**Published:** 2009-01

**Authors:** Lisa M. Koonin, Martin S. Cetron

**Affiliations:** Centers for Disease Control and Prevention, Atlanta, Georgia, USA

**Keywords:** Pandemic, influenza, school closure, letter

## To the Editor

Cowling et al. reported on the effects of school closure in Hong Kong, People’s Republic of China, during March 2008 in response to influenza-related deaths of children (*1*). The influenza epidemic started in January 2008 and peaked in late February, but the 2-week school closure did not begin until March 12. Consequently, the school-based epidemic was on the decline by the time officials closed schools. Other studies have suggested that early school closures can help reduce influenza illness in the community and among school children, especially during a pandemic (*2*–*6*). However, surveillance systems that rely on school absenteeism or deaths would likely provide information too late during the outbreak for school closure to effectively reduce influenza transmission.

The Centers for Disease Control and Prevention (CDC) has recommended early closure of schools as a community mitigation measure in the event of a severe pandemic (*7*). Specifically, CDC recommends rapidly initiating activities such as advising sick persons to stay home, dismissing children from schools, closing childcare facilities, and initiating further social distancing measures within a state or a community at the beginning of the upslope of a pandemic wave (acceleration interval), i.e., when cases are initially identified and community transmission begins to occur (*8*). We concur with the authors that the 2007–08 influenza season was already waning by the time the decision was made to close schools (deceleration interval).

School closure used as a single pandemic control measure is predicted to be less effective than early, concurrent use of multiple measures. Socially disruptive measures like early school closure and keeping children from congregating in the community would likely reduce community transmission of pandemic disease, but would also create secondary challenges (*9*,*10*). Therefore, to ensure maximal benefit for reducing disease transmission, interventions should be implemented early and concomitantly with other nonpharmaceutical and pharmaceutical measures, accompanied by public education, and used judiciously based on pandemic severity.
