# Supplementary material for: School Closure to Reduce Influenza Transmission
Source: Emerg Infect Dis. 2009 Jan;15(1):137–8. doi: 10.3201/eid1501.081289 (PMC2660715; doi:10.3201/eid1501.081289)
Supplement: Related texts

# Reply: In Response

**Authors:** Benjamin J. Cowling, Eric H.Y. Lau, Gabriel M. Leung

**Affiliations:** The University of Hong Kong, Hong Kong Special Administrative Region, People's Republic of China

We agree with Koonin and Cetron (*1*) that early application of any intervention during an influenza epidemic or pandemic is critical in maximizing population health benefits. Further, the longer an intervention is sustained, the greater the likely benefit.

Whether surveillance data can inform public health interventions may depend on the timeliness of the data as well as the length of the epidemic. In tropical and subtropical settings, influenza tends to circulate longer. Although duration of the epidemic could enable delayed interventions a chance of success, social distancing interventions may need to be sustained to ensure that the epidemic does not revive when the intervention period ends.

One important study not mentioned by Koonin and Cetron is a natural experiment in France where the staggering of school holiday periods in different regions enabled Cauchemez et al. to estimate that school holidays prevent 16%–18% of seasonal influenza cases (*2*). In contrast to our study of a single school closure event in response to 1 seasonal outbreak, the French study considered preplanned holiday periods spanning many years.

Although pandemic plans often describe action to be taken depending on features in the epidemic curve (e.g., the acceleration interval as the upslope of the epidemic curve), we would argue that more focus should be given to underlying transmission dynamics. In our analysis of the effect of school closures in Hong Kong, we used a simple statistical technique (*3*) to estimate the underlying reproductive number. Changes in the epidemic curve may lag behind changes in the underlying transmission dynamics by at least 1 serial interval, as has previously been shown for severe acute respiratory syndrome (*3*–*5*). Public health practitioners must be encouraged to use these methods routinely.

Finally, we concur that a multipronged, targeted, layered approach will likely provide the best mitigation strategy in the event of a pandemic. However, we caution against conflating good public health practice of “pulling out all the stops” in the event of a pandemic with good scientific practice of evaluating the independent effect of school closures, which was the object of our article.

## References

[1] Koonin LM, Cetron MS. School closure to reduce influenza transmission. Emerg Infect Dis. 2009;15:137–8. 10.3201/eid1501.08128919116082PMC2660715

[2] Cauchemez S, Valleron AJ, Boelle PY, Flahault A, Ferguson NM. Estimating the impact of school closure on influenza transmission from sentinel data. Nature. 2008;452:750–4. 10.1038/nature0673218401408

[3] Cowling BJ, Ho LM, Leung GM. Effectiveness of control measures during the SARS epidemic in Beijing—a comparison of the Rt curve and the epidemic curve. Epidemiol Infect. 2008;136:562–6. 10.1017/S095026880700872217568476PMC2870828

[4] Wallinga J, Teunis P. Different epidemic curves for severe acute respiratory syndrome reveal similar impacts of control measures. Am J Epidemiol. 2004;160:509–16. 10.1093/aje/kwh25515353409PMC7110200

[5] Cauchemez S, Boelle PY, Donnelly CA, Ferguson NM, Thomas G, Leung GM, Real-time estimates in early detection of SARS. Emerg Infect Dis. 2006;12:110–3.1649472610.3201/eid1201.050593PMC3293464
